# Prophylactic therapy with human amniotic fluid stem cells improved survival in a rat model of lipopolysaccharide-induced neonatal sepsis through immunomodulation via aggregates with peritoneal macrophages

**DOI:** 10.1186/s13287-020-01809-1

**Published:** 2020-07-20

**Authors:** Yu Sato, Daigo Ochiai, Yushi Abe, Hirotaka Masuda, Marie Fukutake, Satoru Ikenoue, Yoshifumi Kasuga, Masayuki Shimoda, Yae Kanai, Mamoru Tanaka

**Affiliations:** 1 Department of Obstetrics and Gynecology, Keio University School of Medicine, 35, Shinanomachi Shinjyukuku, Tokyo, 160-8582 Japan; 2 Department of Pathology, Keio University School of Medicine, Tokyo, Japan

**Keywords:** Human amniotic fluid stem cells, Neonatal sepsis, Inflammatory cytokines, Macrophage polarization, Immunomodulation, Mesenchymal stem cells

## Abstract

**Background:**

Despite recent advances in neonatal care, sepsis remains a leading cause of mortality in neonates. Mesenchymal stem cells derived from various tissues, such as bone marrow, umbilical cord, and adipose tissue, have beneficial effects on adult sepsis. Although human amniotic fluid stem cells (hAFSCs) have mesenchymal stem cell properties, the efficacy of hAFSCs on neonatal sepsis is yet to be elucidated. This study aimed to investigate the therapeutic potential of hAFSCs on neonatal sepsis using a rat model of lipopolysaccharide (LPS)-induced sepsis.

**Methods:**

hAFSCs were isolated as CD117-positive cells from human amniotic fluid. Three-day-old rat pups were intraperitoneally treated with LPS to mimic neonatal sepsis. hAFSCs were administered either 3 h before or at 0, 3, or 24 h after LPS exposure. Serum inflammatory cytokine levels, gene expression profiles from spleens, and multiple organ damage were analyzed. hAFSC localization was determined in vivo. In vitro LPS stimulation tests were performed using neonatal rat peritoneal macrophages co-cultured with hAFSCs in a cell-cell contact-dependent/independent manner. Immunoregulation in the spleen was determined using a DNA microarray analysis.

**Results:**

Prophylactic therapy with hAFSCs improved survival in the LPS-treated rats while the hAFSCs transplantation after LPS exposure did not elicit a therapeutic response. Therefore, hAFSC pretreatment was used for all subsequent studies. Inflammatory cytokine levels were elevated after LPS injection, which was attenuated by hAFSC pretreatment. Subsequently, inflammation-induced damages in the brain, lungs, and liver were ameliorated. hAFSCs aggregated with peritoneal macrophages and/or transiently accumulated in the liver, mesentery, and peritoneum. Paracrine factors released by hAFSCs induced M1-M2 macrophage polarization in a cell-cell contact-independent manner. Direct contact between hAFSCs and peritoneal macrophages further enhanced the polarization. Microarray analysis of the spleen showed that hAFSC pretreatment reduced the expression of genes involved in apoptosis and inflammation and subsequently suppressed toll-like receptor 4 signaling pathways.

**Conclusions:**

Prophylactic therapy with hAFSCs improved survival in a rat model of LPS-induced neonatal sepsis. These effects might be mediated by a phenotypic switch from M1 to M2 in peritoneal macrophages, triggered by hAFSCs in a cell-cell contact-dependent/independent manner and the subsequent immunomodulation of the spleen.

## Background

Despite recent advances in neonatal intensive care, systemic inflammation such as sepsis is still a leading cause of mortality and morbidity in preterm infants, particularly in those with extremely low birth weights [1, 2]. Preterm neonates are more vulnerable to infectious diseases leading to higher sepsis-related mortality compared to adults due to the neonatal immune response being quantitatively and qualitatively distinct from that of adults [3, 4]. Lacking a fully developed adaptive immune system, newborns must rely on the innate immune response for protection against infection [3, 5]. In addition, low numbers of lymphocytes in neonates exacerbate the excessive production of pro-inflammatory cytokines against infection [3, 6]. Therefore, macrophages are considered important initiators and regulators of the innate immune response in neonates.

Mesenchymal stem cells (MSCs) possess unique paracrine and immunosuppressive properties, which make them useful candidates for cellular therapy [7–9]. In particular, numerous preclinical studies have successfully used MSCs to improve outcomes in adult animal models of sepsis and organ injury [7, 10–12], and clinical studies to test their potential are ongoing in adults [13, 14]. However, the distinct differences in immune responses between neonates and adults have been reported in rodents and humans [3, 6], and little is known about the therapeutic effect of MSCs on neonatal sepsis [15].

Among MSCs, human amniotic fluid stem cells (hAFSCs) offer the intriguing potential for autologous MSC treatment for a variety of complications in neonates, including congenital abnormalities and preterm birth [16]. Recently, we reported that hAFSC treatment attenuated local inflammation in rodent models of perinatal diseases such as hypoxic-ischemic encephalopathy [17] and fetal myelomeningocele [18]. hAFSCs generated during pregnancy could be potentially used for autologous cell therapy treatment in neonates, if required immediately after birth or during pregnancy [19]. However, there is no report on the therapeutic efficacy of hAFSCs for the treatment of neonatal sepsis.

The aim of this study is to determine the effect of hAFSC transplantation in a rat model of LPS-induced neonatal sepsis.

## Methods

### Isolation, culture, and immunophenotypic characterization of CD117^+^ amniotic fluid cells

The study was approved by the Institutional Review Board of Keio University School of Medicine (no. 20140285), and informed consent was obtained from all the volunteer donors. Five-milliliter amniotic fluid samples were obtained from two pregnant women who underwent amniocentesis at 15 and 16 weeks of gestation. CD117-positive (CD117^+^) cells were isolated as hAFSCs, as described previously [17–22]. Briefly, within 2 h, the samples were centrifuged at 200×*g* for 5 min. After removing the supernatant, the cell pellet was cultivated in growth medium comprising alpha modified Eagle minimum essential medium (α-MEM; Invitrogen, Carlsbad, CA), 15% fetal bovine serum (FBS) (Invitrogen), 1% L-glutamine (Invitrogen), 1% penicillin/streptomycin (Invitrogen), and 40% AmnioMax-II (Life Technologies, Carlsbad, CA). After the cell population became sub-confluent, the cells were counted, and the CD117^+^ cells were isolated as hAFSCs using a magnetic cell sorting kit (Miltenyi Biotec, Auburn, CA).

CD117^+^ cells were characterized by flow cytometry for surface markers, as described in our previous studies [17, 18, 21]. The antibodies used for flow cytometry are listed in Table S1. CD117^+^ cells were cultured in “adipogenic differentiation medium” and “osteogenic differentiation medium” (PromoCell, Heidelberg, Germany) according to the manufacturer’s protocol. To induce chondrogenic differentiation, a total of 1.0 × 10^6^ cells were seeded in EZSPHERE (AGC Techno Glass, Tokyo, Japan), then cultured for 12 days in “chondrogenic differentiation medium” (PromoCell). CD117^+^ cells were also characterized by real-time polymerase chain reaction (RT-qPCR) for the expression of molecular differentiation markers into adipogenic, osteogenic, or chondrogenic lineages. RT-qPCR was performed in duplicate in a volume of 25 μL per reaction using a 96-well Bio-Rad CFX96 Real-Time PCR System (Bio-Rad, Richmond, CA). Reaction mixtures contained 5 ng genomic DNA as the template, 0.4 mM of each primer (FASMAC, Atsugi, Kanagawa, Japan), SYBR Premix Ex Taq II (Tli RNaseH Plus; Takara Bio), and sterile H_2_O. The primer sets are listed in Table S2. We analyzed the relative gene expression in each sample by the 2^−ΔΔCT^ method. Gene expression values were normalized to *β-actin* levels as an internal control.

### Animals

All experiments were approved by the Animal Committee of Keio University (no. 18003-0). At postnatal day 3 (P3), Sprague Dawley (SD) male rat pups (Charles River Laboratories Japan Inc., Kanagawa, Japan) were randomly assigned to three experimental groups. These groups were treated with intraperitoneal (i.p.) injections (lower abdomen, both sides) as follows: control group (saline NaCl 0.9%), LPS group (LPS; *Escherichia coli* O55: B5, Sigma-Aldrich, Steinheim, Germany), and hAFSCs+LPS group. LPS 0.25 mg/kg dissolved in 50 μL [23], 1.0 × 10^6^ hAFSCs dissolved in 50 μL saline, or 50 μL saline was injected intraperitoneally in the rats. The optimal timing of hAFSCs administration was investigated by screening the effect at four time points (3 h before, 0 h, 3 h, and 24 h after LPS exposure). For survival analysis, rats were monitored 6, 12, 24, and 48 h after LPS administration and the survival checks were continued once a day up to 30 days after LPS administration. We investigated another group that received only hAFSCs (hAFSCs group) (*n* = 39) for the assessment of the negative effects of hAFSCs administration. We also monitored the survival in the hAFSCs+LPS group using different donor-derived hAFSC cell lines.

**Fig. 1:**
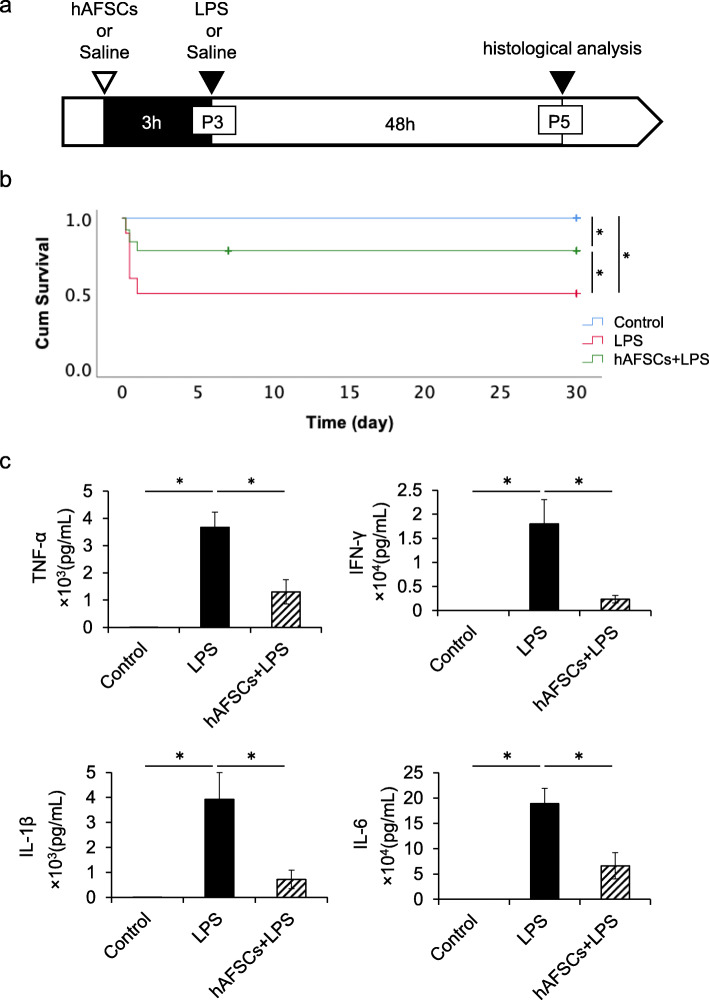
hAFSCs improved mortality and serum cytokine levels in LPS-induced neonatal sepsis. **a** Timecourse of the experimental protocol. Three-day-old rat pups were injected LPS intraperitoneally. Three hours before LPS exposure, rats were treated with hAFSCs or saline. **b** Survival rate until 30 days after LPS administration in control (*n* = 30), LPS (*n* = 40), and hAFSCs+LPS group (*n* = 28). hAFSCs treatment significantly improved survival compared to saline-treated animals after LPS exposure. **c** Levels of the pro-inflammatory cytokine TNF-α, IFN-γ, IL-1β, and IL-6 in serum in the control (*n* = 5), LPS (*n* = 7), and hAFSCs+LPS group (*n* = 6). Results are presented as mean ± SEM. *p < 0.05

Based on the results from the screening studies, all subsequent studies were conducted by hAFSC pretreatment 3 h before LPS exposure (Fig. 1a), as only this group showed any significant therapeutic effect (Table 1).

**Table 1:** The optimal timing of hAFSC administration for eliciting significant therapeutic effect in LPS-induced neonatal sepsis

Timing of hAFSC injection	Survival rate (%)	*N*	*p* value
LPS only	50.0	40	–
− 3 h	78.5	28	0.015*
0	56.3	16	0.67
+ 3 h	50.0	18	1.00
+ 24 h	50.0	20	1.00

The chi-squared test between the LPS only and each of the other groups

*p < 0.05

### Analysis of serum inflammatory cytokines and levels of organ function indicators

For analysis of cytokines and organ function indicators in serum, blood was collected from the heart at 6 h after LPS administration [23, 24]. Serum was prepared in uncoated tubes by centrifugation at 4000 rpm for 5 min and stored at − 80 °C until analysis. Serum levels of tumor necrosis factor (TNF)-α, interferon (IFN)-γ, interleukin (IL)-1β, and IL-6 were determined 6 h after LPS administration using MILLIPLEX® Multiplex Assays using Luminex® with a rat cytokine/chemokine panel (Merck Millipore, Billerica, MA, USA) according to the manufacturer’s protocol [24]. The MILLIPLEX® plate was read with a Luminex®200 xPONET® system. Data were analyzed using the xPONENT® software (Luminex, Austin, TX, USA). Serum levels of hepatic dysfunction indicators: aspartate aminotransferase (AST) and alanine aminotransferase (ALT) were analyzed 48 h after LPS administration (SRL, Tokyo, Japan) [24, 25]. To assess whether hAFSC administration induces hyperinflammation, we also evaluated TNF-α levels in serum by enzyme-linked immunosorbent assay (ELISA); Rat TNF-alpha Quantikine ELISA Kit® (R&D Systems, Minneapolis, MN).

### Immunohistochemical analysis

The brain, lung, and liver were harvested 48 h after LPS injection. Excised specimens were fixed with 4% paraformaldehyde for paraffin embedding. Paraffin sections (4 μm) were subjected to hematoxylin-eosin (H&E), periodic acid-Schiff (PAS) staining, and immunohistochemistry. The white matter around the hippocampus was assessed in the brain sections [26]. Astrocytes were evaluated by anti-glial fibrillary acidic protein (GFAP) antibodies (Dako Corporation, Carpinteria, CA) visualized by Vectastain ABC Kit (Vector Laboratories, Burlingame, CA, USA). Microglial cells were assessed by rabbit anti-ionized calcium-binding adapter molecule 1 (Iba-1; Wako, Osaka, Japan, 1:100), and nuclei were counterstained with Hoechst (Wako, Osaka, Japan, 1:100). Immunohistochemistry was performed using the rabbit anti-myeloperoxidase (MPO; Abcam, Cambridge, UK, 1:50) antibody, or mouse anti-Iba-1 (Iba-1; Wako, Osaka, Japan, 1:500) and the nuclei were counterstained with Hoechst (Wako), in all other organ samples. Antibodies used for immunohistochemistry are listed in Table S3. Images were captured using a BZX-810 camera (Keyence, Osaka, Japan), and morphometric analysis was performed using ImageJ software (www.rsb.info.nih.gov/ij). To investigate neuroinflammation, we counted GFAP-positive cells and Iba-1 positive cells in the brain sections [26]. To evaluate neutrophil infiltration, we determined the percentage of MPO-positive cells in the lung and liver [27]. Thereafter, we counted the Iba-1 positive cells to evaluate macrophage activation (Fig. 3). Lung injury was determined by radial alveolar count (RAC) and mean linear intercepts (MLI) using the ImageJ software [28–30].

### hAFSC tracking after intraperitoneal application

hAFSCs were labeled with the fluorescent tracer XenoLight DiR (Xenogen Corporation, Caliper Life Sciences, Alameda, CA) following the manufacturer’s protocol [31]. DiR-labeled hAFSCs were administered intraperitoneally at P3 3 h before LPS injection. The organs were harvested 12, 24, 48, 72 h, and 7 days after DiR-labeled hAFSC administration. The collected organs were imaged using the IVIS® Spectrum (Caliper Life Sciences). Filter conditions and illumination settings for DiR imaging were set as 710/760 nm (excitation/emission), high lamp level, medium binning, filter 1, and 1.0 s exposure time. Grayscale and fluorescent images of each organ were analyzed using Living Image software version 4.3 (Xenogen).

Macroscopically, the cellular aggregates appeared as small clusters, which varied in the number and the size of the cluster. Microscopic analysis of a single aggregate was carried out using an anti-human mitochondria antibody (Sigma-Aldrich, St. Louis, MO) for the hAFSCs and an anti-CD68 antibody (Bioss antibodies, Woburn, MA) for the peritoneal macrophages. Antibodies used for immunocytochemistry are listed in Table S3. Images were captured using a BZX-810 camera (Keyence).

### Analysis of LPS-stimulated macrophages co-cultured with hAFSCs

Peritoneal macrophages were obtained, as previously described [32]. Briefly, rat peritoneal exudate cells were elicited by intraperitoneal injection with 2 mL of 3% sterile sodium thioglycolate (BD, Franklin Lakes, New Jersey) in SD male rat pups (P3). Peritoneal cells were obtained 3 days later by peritoneal lavage with cold PBS and washed in cold RPMI medium. A minimum of 25% of the cells were macrophages as determined by flow cytometry analysis using FITC Mouse Anti-Rat CD11b (BD Biosciences) according to the manufacturer’s protocol.

To determine whether hAFSCs could regulate the secretion of pro-inflammatory cytokine in LPS-stimulated macrophages in a cell-cell contact independent/dependent manner, the two types of cells were co-cultured either in a transwell (0.4 μm pore size; Costar; Corning, NY) system or a standard well, and then stimulated with LPS. CD11b-positive macrophages were incubated with RPMI 1640 containing 10% FBS and 1% penicillin/streptomycin. To evaluate the effects of co-culture with cell-cell contact, peritoneal macrophages and hAFSCs were co-incubated in the presence of LPS (0.1 μg/mL) for 4 h at 37 °C in a standard 24-well plate (Costar®; Corning). To test the effects of co-culture without cell-cell contact, the macrophages (1 × 10^5^ cells per well) were placed in the upper insert of a transwell system (0.4 μm pore, Corning), and hAFSCs (1 × 10^6^ cells) were placed in the lower well. The study groups included the following: control group, LPS group, hAFSCs+LPS (no contact between macrophage and hAFSCs) group, and hAFSCs+LPS (cell-cell contact between macrophage and hAFSCs) group (Fig. 4a).

Cell-free supernatants and macrophage RNA were collected 4 h after LPS stimulation. RNA was extracted using the RNeasy Mini Kit (Qiagen), and reverse transcription of total RNA was performed using the Prime Script RT Master Mix (Takara Bio Inc., Shiga, Japan). M1 (TNF-α) and M2 (IL-10) marker levels in the supernatants were determined by ELISA using Rat TNF-alpha Quantikine ELISA Kit® and Rat IL-10 Quantikine ELISA Kit® (R&D Systems), and gene expressions of M1 (TNF-α and IL-1β) and M2 (IL-10 and Arginase-1) markers were assessed by RT-qPCR. Gene expression values were normalized to *β-actin* levels as an internal control. The primer sets are listed in Table S2. Additionally, gene expressions were assessed using two different donor-derived hAFSC cell lines.

### DNA microarray analysis of the spleen

Six hours after LPS administration, total spleen RNA was extracted using the RNeasy Mini Kit (Qiagen, Hilden, Germany). Genome-wide expression analysis was performed using the total RNA, which was labeled and hybridized to GeneChip® Clariom S array, Rat (Affymetrix, Santa Clara, CA). Principal component analysis (PCA) was performed before the analyses of each sample. Gene expression patterns were compared between the control group, LPS group, and hAFSCs+LPS group. Once genes with significant differences in expression were identified, fold changes were calculated between the LPS group and hAFSCs+LPS group [33]. The pathway analysis was performed using WikiPathways.

### Statistical analysis

All values were expressed as mean ± standard error. Statistical differences between groups were assessed using analysis of variance and Tukey’s honest significant difference.

The chi-squared test and log-rank test were used for comparing survival data. Statistical analyses were performed using JMP14.0 software (SAS Institute, Cary, NC). *P* values less than 0.05 were considered statistically significant.

## Results

### Isolation, culture, and immunophenotypic characterization of hAFSCs

CD117^+^ amniotic fluid cells were isolated using a magnetic cell sorting kit. After immunoselection and passage in culture, spindle-shaped cells were expanded as stable lines (Additional file 1: Fig. S1a). Markers of cell surface antigens on hAFSCs were evaluated by flow cytometry. hAFSCs were positive for mesenchymal markers (CD73, CD90, and CD105) and negative for hematological markers (CD14, CD34, and CD45) (Additional file 1: Fig. S1b). We also determined the differentiation capability of CD117^+^ amniotic fluid cells. These cells could differentiate toward adipogenic, osteogenic, and chondrogenic lineages, as shown by the expression of the respective molecular markers (Additional file 1: Fig. S1c).

### Prophylactic therapy with hAFSCs improved survival in LPS-induced neonatal sepsis

P3 rat pups were treated with hAFSCs dissolved in saline or saline alone at either 3 h before, 0 h, 3 h, or 24 h after LPS exposure (Table 1). The survival rate of the LPS group was 50.0%, which was significantly increased by up to 78.5% by hAFSC pretreatment (Table 1). However, no therapeutic effect was observed when hAFSCs were administered after LPS treatment. Treatment with hAFSCs alone did not affect the survival rate (100%). All pups surviving at 48 h after LPS administration could survive for a longer period (Fig. 1b). There were no significant differences between the therapeutic effects of the two hAFSC cell lines (Additional file 2: Fig. S2a).

### hAFSCs reduced pro-inflammatory cytokines in serum after LPS administration

We investigated the pro-inflammatory cytokines, TNF-α, IFN-γ, IL-1β, and IL-6 in serum 6 h after LPS administration. There were significant elevations in the concentrations of all cytokines in the serum of the LPS group compared to those of the control group. hAFSC pretreatment significantly attenuated the LPS-stimulated increase in cytokine levels (Fig. 1c). On the other hand, treatment with hAFSCs alone did not affect the levels of inflammatory cytokines in rats, as demonstrated by the levels of TNF-α in the study groups (control group, LPS group, hAFSCs+LPS group, and hAFSCs group) (Additional file 3: Fig. S3).

### hAFSCs attenuated multiple organ dysfunction following inflammation

**Fig. 2:**
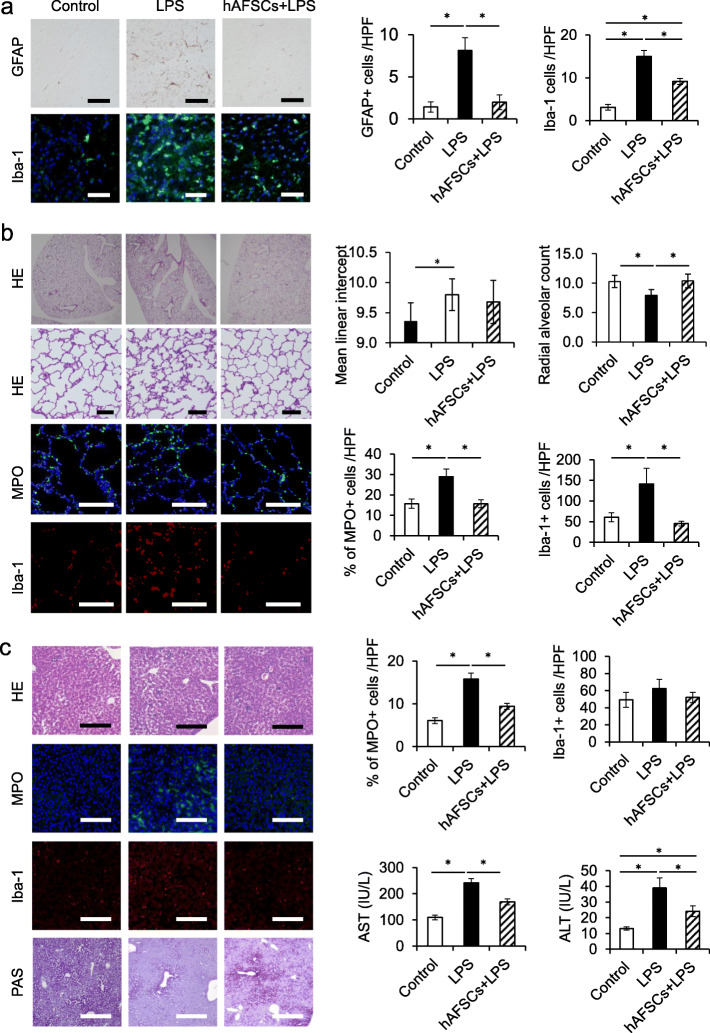
hAFSCs attenuated inflammation in the brain, lung, liver, and kidney after LPS challenge. Histology of the brain, lung, and liver tissues from the control (*n* = 6), LPS (*n* = 8), and hAFSCs+LPS (*n* =8) 48 h after LPS exposure. **a** Representative image of brains. GFAP staining, upper row (scale bars, 50 μm); and Iba-1 staining, lower row (scale bars, 50 μm). The graphs showed the number of GFAP-positive cells/HPF and Iba-1-positive cells/HPF in each group. **b** Representative image of the lungs. H&E staining, upper row (scale bars, 100 μm); MPO staining, middle row (scale bars, 50 μm); and Iba-1 staining, lower row (scale bars, 50 μm). Radial alveolar counts and mean linear intercepts were performed by averaging seven measurements per rat. The other graphs in the lower row show the percentage of MPO-positive cells/HPF and the number of Iba-1-positive cells/HPF in each group. **c** Representative image of livers. H&E staining, the first row (scale bars, 100 μm); MPO staining, the second row (scale bars, 50 μm); Iba-1 staining, the third row (scale bars, 50 μm); and PAS staining, the last row (scale bars, 200 μm). The graphs in the upper row show the percentage of MPO-positive cells/HPF and the number of Iba-1-positive cells/HPF in each group. The graphs in the lower row show the levels of aspartate aminotransferase (AST) and alanine aminotransferase (ALT) in serum in each group 48 h after LPS exposure. Results are presented as mean ± SEM. *p < 0.05

LPS administration induced neuroinflammation in the brain, as indicated by the presence of the GFAP-positive cells and Iba-positive cells. However, hAFSCs pretreatment significantly reduced neuroinflammation (Fig. 2a). Likewise, in the lungs and liver, LPS induced tissue inflammation, as indicated by the presence of MPO-positive cells and Iba-positive cells. Inflammation-induced tissue damage after LPS administration was indicated by RAC and MLI in the lungs and by glycogen storage capacity and serum AST and ALT in the liver. hAFSC pretreatment significantly attenuated tissue inflammation induced by LPS and subsequently ameliorated organ dysfunctions (Fig. 2b, c).

### hAFSCs were transiently localized in the liver and mesentery

**Fig. 3:**
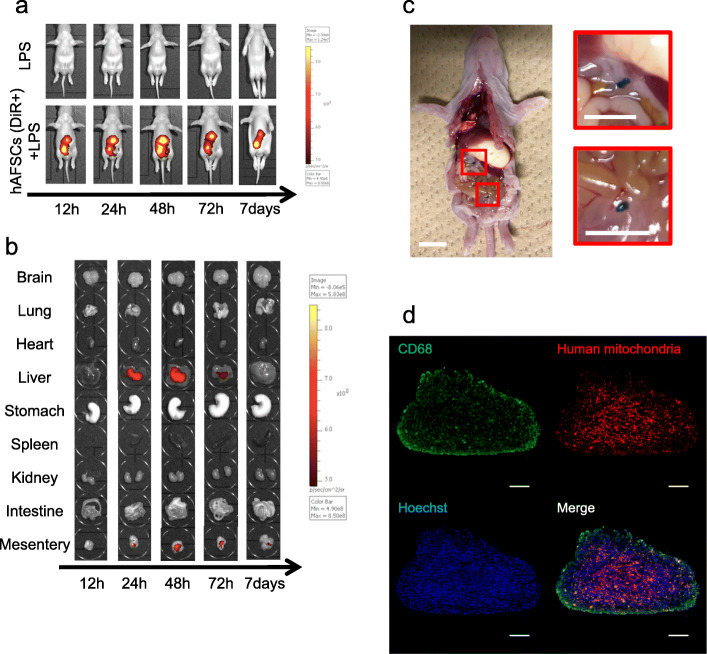
Distribution of hAFSCs after intraperitoneal transplantation. Distribution of hAFSCs after intraperitoneal injection. The distribution of hAFSCs (*n* = 3) after injection was detected by in vivo imaging (IVIS®). Cells were labeled with DiR and injected 3 h before hAFSCs injection. **a** The representative pictures from the ventral side. **b** Pictures and graphs of each organ in the time-course of distribution in LPS and hAFSCs+LPS groups. **c** In vivo image in the peritoneal cavity 48 h after LPS administration. DiR-labeled hAFSCs were aggregated as *white dashed lines* outlines (scale bars, 10 mm). **d** Microscopic analysis of a single aggregate showed that the cellular aggregates were mainly composed of human mitochondria-positive hAFSCs surrounded by CD68^+^ peritoneal macrophages (scale bars, 100 μm)

To study the fate of hAFSCs after intraperitoneal injection, we tracked DiR-labeled hAFSCs administered intraperitoneally, using an in vivo imaging system (IVIS® Spectrum). The fluorescence accumulated in the abdominal cavity (Fig. 3a) and DiR-labeled hAFSCs migrated to and were transiently localized in the liver and mesentery within 72 h after administration, although the fluorescence intensity of hAFSCs gradually decreased (Fig. 3b). These findings indicated that hAFSC pretreatment attenuated inflammation-induced dysfunctions even in organs where hAFSCs have barely reached, such as the brain and lung.

### hAFSCs assemble with peritoneal macrophages in the peritoneal cavity

We observed cellular aggregates in the peritoneal cavity of rats that received hAFSCs. Macroscopically, the aggregates appeared as small clusters, which varied in the number and the size of clusters (Fig. 3c). Microscopic analysis of single aggregates showed that the cellular aggregates were mainly composed of human mitochondria-positive hAFSCs and CD68^+^ peritoneal macrophages (Fig. 3d).

### hAFSCs induced a macrophage phenotypic switch from M1 to M2 in both cell-cell contact-independent and contact-dependent manners at the transcriptional level

**Fig. 4:**
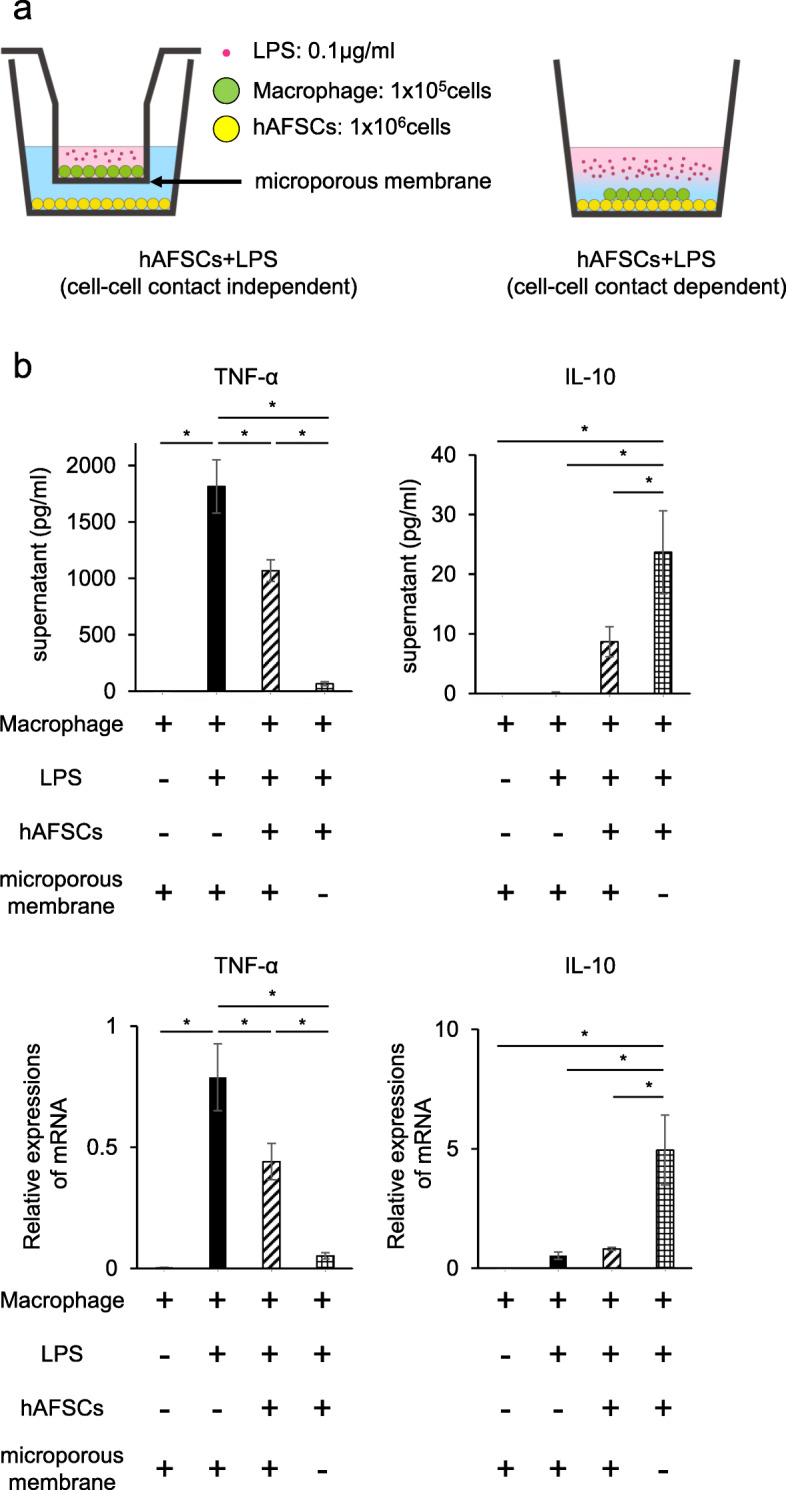
hAFSCs induced a macrophage phenotypic switch from M1 to M2 in a cell-cell contact-independent and dependent manner at the transcriptional level. **a** The schema of the culture methods. hAFSCs and peritoneal macrophages were co-cultured in the presence of LPS (right schema). Transwell inserts were used in wells to prevent cell-cell contact between hAFSCs with macrophages (left schema). **b** TNF-α in the supernatant in each well 4 h after LPS exposure was measured by ELISA (*n* = 8) (upper left row). mRNA expression of TNF-α was investigated by RT-qPCR (*n* = 8) (lower left row). IL-10 in the supernatant of each well 4 h after LPS exposure was measured (*n* = 8) (upper right row). mRNA expression of IL-10 was investigated by RT-qPCR (*n* = 8) (lower right row). Results are presented as mean ± SEM. *p < 0.05

M1/M2 polarization of macrophages regulates the inflammation and regeneration process [34]. To explore the effect of hAFSCs on peritoneal macrophages, we examined TNF-α secreted from macrophages as an M1 marker and IL-10 as an M2 marker (Fig. 4b). TNF-α levels secreted from macrophages after LPS administration was significantly reduced by hAFSCs in a cell-cell contact-independent manner. Cell-cell contact between the macrophages and hAFSCs further reduced the TNF-α level (Fig. 4b). Also, TNF-α reduction was induced by cell-cell contact at the transcriptional level (Fig. 4b). Alternatively, the IL-10 level in supernatants was radically increased by the cell-cell contact between LPS-stimulated macrophages and hAFSCs (Fig. 4b). These changes were also regulated at the transcriptional level.

In addition, these phenotypic changes were confirmed by IL-1β and Arginase-1 as M1 and M2 markers, respectively (Additional file 4: Fig. S4). Further, both the hAFSC cell lines tested had similar effects on macrophage polarization (Additional file 2: Fig. S2b).

### hAFSC administration reduced the expression of genes involved in inflammation and apoptosis in the spleen

**Fig. 5:**
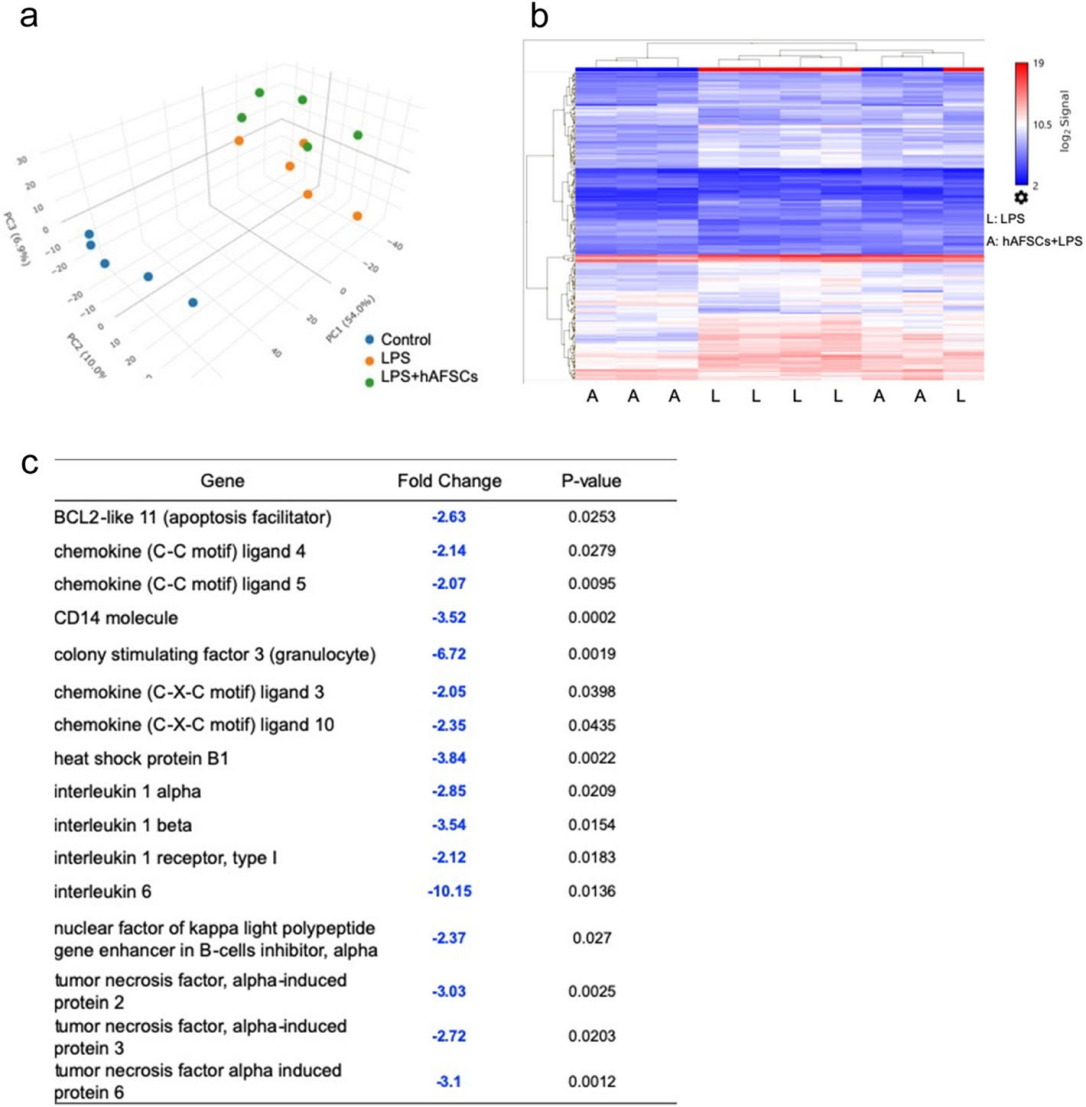
hAFSCs reduced the expression of apoptotic and inflammatory genes in the spleen. **a** The PCA mapping of each group; (1) control, (2) LPS, and (3) hAFSCs+LPS (*n* = 5). **b** Heat map shows the gene expression patterns of the LPS group and the hAFSCs+LPS group. **c** Fold changes of selected immune-related genes in the hAFSCs+LPS group compared with those in the LPS group

The spleen regulates systemic immune responses of the whole body [35]. To determine the immune responses to LPS administration, we performed DNA microarray analysis on spleen tissue. The patterns of PCA mapping in the spleen demonstrated that gene expressions were significantly changed by LPS administration and that prophylactic treatment with hAFSCs modulated the gene expression (Fig. 5a). There were 228/23,188 genes differentially expressed between the LPS group and the hAFSCs+LPS group (Fig. 5b). We focused on genes that exhibited significant differences in expression between the LPS group and the hAFSCs+LPS group (*p* < 0.05) in the DNA microarray analysis (Fig. 5c). LPS treatment upregulated the expressions of genes involved in apoptosis and inflammation. hAFSC pretreatment attenuated the upregulation of the genes involved in apoptosis such as *BCL2-like 11* and those involved in inflammation such as *CC* or *CXC chemokine ligand*, *colony-stimulating factor 3*, *IL-1* and *6*, and *TNF-α-induced proteins*.

### hAFSC administration suppressed the Toll-like receptor signaling pathway and cytokines and inflammatory response pathway in the spleen

The WikiPathways database was used to annotate the differentially expressed genes, and they were identified to be involved in the Toll-like receptor (TLR) signaling pathway (https://www.wikipathways.org/index.php/Pathway:WP1309) and cytokine and inflammatory response pathway (https://www.wikipathways.org/index.php/Pathway:WP271).

The genes that were downregulated in the hAFSCs+LPS group compared to that in the LPS group (negative log2 fold change) are shown in shades of green, and those that were upregulated are shown in shades of red (Fig. S5 and S6). hAFSC pretreatment downregulated TLR4 signaling and inhibited the expression of inflammatory cytokines such as *Tnf*, *Il-1b*, *Il-6*, and *Ccl5* by modulating the expression of *cd14*, *Nfkb family*, and *Jun/Fos* in the spleen (Additional file 5: Fig. S5). Also, hAFSCs generally suppressed the expression of inflammatory genes, including *Csf3*, *Il-1a*, *Il-1b*, *Il-6*, and *Tnf* in the macrophages (Additional file 6: Fig. S6).

## Discussion

In this study, we established a rat model of neonatal sepsis by LPS administration into rat pups, particularly mimicking neonatal sepsis of preterm infants in humans, and demonstrated the therapeutic effects of prophylactic treatment with hAFSCs on neonatal sepsis using our model. Specifically, prophylactic treatment with hAFSCs suppressed systemic inflammation and multiple organ dysfunction and improved the survival rate in LPS-induced neonatal rat sepsis. These effects might be mediated by the phenotypic switch of peritoneal macrophages from M1 to M2, which was induced by hAFSCs both in a cell-cell contact-dependent and contact-independent manner, and the subsequent immunomodulation of the spleen.

To date, rodent models have been used extensively to investigate the physiological process of sepsis [11]. In systemic challenge models, bacteria (i.e., *E. coli*) or bacteria-derived toxins (i.e., LPS) are administered into adult rodents [7, 11]. However, the distinct differences in immune responses between neonates and adults have been reported in both rodents and humans, possibly contributing to the higher mortality observed in neonates compared to adults [3, 4]. It has been reported that neonatal rodents were hyper-susceptible to LPS in an age-dependent manner [6, 33]. Consequently, 3- to 5-day-old rodents have been utilized to mimic the immune response of human preterm infants [36, 37]. We demonstrated that hAFSC pretreatment improved the survival rate in neonatal rat sepsis from 50 to 80%, following the reduction of pro-inflammatory cytokine levels in serum after LPS administration (Fig. 1). Zhu et al. revealed that MSCs derived from the human umbilical cord significantly improved survival in *E. coli*-induced neonatal rat sepsis [15], which is consistent with our findings. In addition to the high mortality rate, sepsis presents an increased level of inflammatory activation, which results in multiple organ impairments. In the present study, hAFSC pretreatment improved tissue inflammation and attenuated LPS-induced tissue damages in the lung, liver, and brain as determined by the histological analysis (Fig. 2), which is in agreement with the demonstrated therapeutic effects of other MSCs [7, 10, 15, 25, 26].

It has been demonstrated that hAFSCs have the potential to reduce local inflammation in rodent models of perinatal diseases mainly via paracrine factors secreted from hAFSCs locally transplanted or migrated to the damaged tissue regions [16–18, 38–40]. In contrast, our study showed that prophylactic treatment with hAFSCs could systematically reduce the inflammatory damages in the whole body. hAFSCs injected into peritoneal cavity aggregated with peritoneal macrophages and formed spheroid, or migrated to and transiently accumulated in the liver, mesentery, and peritoneum (Fig. 3). Consequently, few hAFSCs were detected in the lung and brain where the therapeutic effects of hAFSCs were observed. These results were consistent with those of the previous study on MSCs using a colitis model [8, 41, 42]. Thus, hAFSCs could protect multiple organs from severe inflammation by adapting an immune regulatory and regeneration-supporting status in the whole body.

**Fig. 6:**
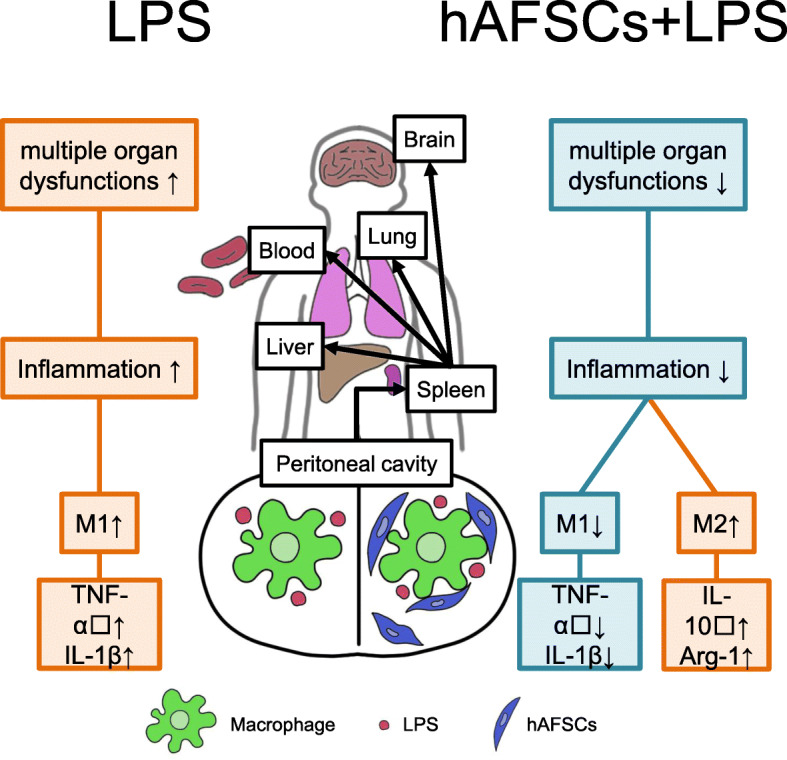
hAFSCs regulated immune reaction by two steps: peritoneal macrophages and spleen. hAFSCs regulated immune reaction in the whole body by two steps—phenotypic switch of peritoneal macrophages from M1 to M2 and the immune reactions of the spleen following the phenotypic switch of peritoneal macrophages

There are two possible steps of immune regulation in the whole body. One is a phenotypic switch of peritoneal macrophage from M1 to M2; the other is immune reactions of the spleen following a phenotypic switch of peritoneal macrophages (Fig. 6). Macrophages represent the majority of immune cells, and lymphocytes are fewer in neonates compared to adults [3, 6]. Our data indicated that the beneficial effects provided by hAFSCs administration were triggered by their action on peritoneal macrophages. hAFSCs could regulate peritoneal macrophage polarization from M1 to M2 via paracrine factors in a cell-cell contact-independent and/or contact-dependent manner (Fig. 4 and Additional file 4: Fig. S4) [43, 44]. Moreover, hAFSCs spontaneously aggregated in the peritoneal cavity and formed spheroids (Fig. 3c, d). Self-activation of hAFSCs by assembly into aggregates could enhance their beneficial effects [41, 45, 46]. Thus, in our study, hAFSCs could act on peritoneal macrophages as the first responder.

The spleen, the central immune organ, plays a role in regulating systemic immune responses in the whole body [35]. Therefore, we focused on the responses in the neonatal spleen after local immunomodulation via peritoneal macrophage. DNA microarray analysis of the spleen revealed that hAFSC administration reduced the expression of genes involved in apoptosis and inflammation and subsequently suppressed the TLR signaling pathway (Fig. 5). Sepsis can induce apoptosis by the mechanisms related to oxidative stress and inflammation [47–49]. Spleen cells undergo apoptosis leading to immune cell loss and immune cell dysfunction [15]. MSCs are capable of attenuating apoptosis by regulating the anti-apoptotic BCL-2 family proteins [50]. With regard to inflammation, hAFSCs downregulated TLR signaling and inhibited the expression of inflammatory cytokines such as *Tnf*, *Il-1b*, and *Il-6*, which could directly contribute to reducing pro-inflammatory cytokines in the serum and regulating inflammation in the whole body. In this study, the expression levels of multiple regulatory factors controlling TLR signaling, such as *cd14*, *Nfkb family*, and *Jun/Fos*, were significantly reduced by hAFSC pretreatment. These results suggest that hAFSCs could suppress TLR signaling in the spleen in a multimodal way. Furthermore, hAFSCs generally suppressed the expression of inflammatory genes in macrophages in the spleen, which are considered important regulators of the innate immune response in neonates [3–6]. Thus, the spleen plays central roles in regulating immune responses in the whole body after local immunomodulation via peritoneal macrophage.

The timing of hAFSCs transplantation may be important for interpreting our findings. In contrast to the previous reports on MSC treatment in an adult sepsis model [7, 11], in our study, beneficial effects were only observed when hAFSCs were administered before LPS exposure (Table 1). This suggests that the pre-formation of cellular aggregates of hAFSCs and macrophages in the peritoneal cavity might be key to the therapeutic effect, and thus, appropriate timing of hAFSCs administration is critical. From an obstetrician’s point of view, neonatal sepsis frequently occurs in premature newborns following preterm premature rupture of the membranes during pregnancy [51]. If the amniotic fluid could be collected during the preterm premature rupture of the membranes or amniocentesis, autologous hAFSCs could be prepared and administered as “preemptive therapy” before neonatal sepsis develops in high-risk pregnancies.

## Conclusions

In conclusion, this study demonstrated that prophylactic therapy with hAFSCs improved survival in an LPS-induced neonatal sepsis model. These effects might be mediated by local adaptation via peritoneal macrophages, induced by hAFSCs could act in a cell-cell contact-dependent or contact-independent manner, and the subsequent immune modulation of the spleen. These results suggest that prophylactic therapy with hAFSCs could have therapeutic potential for neonatal sepsis.

## Supplementary information

**Additional file 1: Figure S1.** Culture, surface marker expression, and differentiation potential of human amniotic fluid stem cells (hAFSCs).

**Additional file 2: Figure S2.** hAFSCs derived from two donors had similar therapeutic effects in vivo and in vitro.

**Additional file 3: Figure S3.** hAFSCs alone did not increase TNF-α in serum in rats.

**Additional file 4: Figure S4.** Supportive information related to macrophage phenotypic switch from M1 to M2 in cell-cell contact-independent/dependent manner at the transcriptional level.

**Additional file 5: Figure S5.** Toll-like receptor signaling pathway.

**Additional file 6: Figure S6.** The cytokines and inflammatory response pathway.

**Additional file 7: Table S1.** List of antibodies used for flow cytometry in this study. **Table S2.** List of primer sequences used for RT-qPCR in this study. **Table S3.** List of antibodies used for immunohistochemistry in this study.

## Data Availability

All data generated or analyzed during this study are included in this published article.

## Abbreviations

hAFSCs: Human amniotic fluid stem cells
LPS: Lipopolysaccharide
MSCs: Mesenchymal stem cells
α-MEM: Alpha modified Eagle minimum essential medium
FBS: Fetal bovine serum
RT-PCR: Real-time polymerase chain reaction
SD: Sprague Dawley
i.p.: Intraperitoneal
TNF: Tumor necrosis factor
IFN: Interferon
IL: Interleukin
AST: Aspartate aminotransferase
ALT: Alanine aminotransferase
ELISA: Enzyme-linked immunosorbent assay
H&E: Hematoxylin-eosin
PAS: Periodic acid-Schiff
GFAP: Anti-glial fibrillary acidic protein
Iba-1: Ionized calcium-binding adapter molecule 1
MPO: Myeloperoxidase
RAC: Radial alveolar count
MLI: Mean linear intercepts
PCA: Principal component analysis
TLR: Toll-like receptor
