# Supplementary material for: Prophylactic therapy with human amniotic fluid stem cells improved survival in a rat model of lipopolysaccharide-induced neonatal sepsis through immunomodulation via aggregates with peritoneal macrophages
Source: Stem Cell Res Ther. 2020 Jul 20;11:300. doi: 10.1186/s13287-020-01809-1 (PMC7370504; doi:10.1186/s13287-020-01809-1)
Supplement: Supplementary file 1 — Additional file 1: Figure S1. Culture, surface marker expression, and differentiation potential of human amniotic fluid stem cells (hAFSCs). [file 13287_2020_1809_MOESM1_ESM.pptx]

## Slide 1
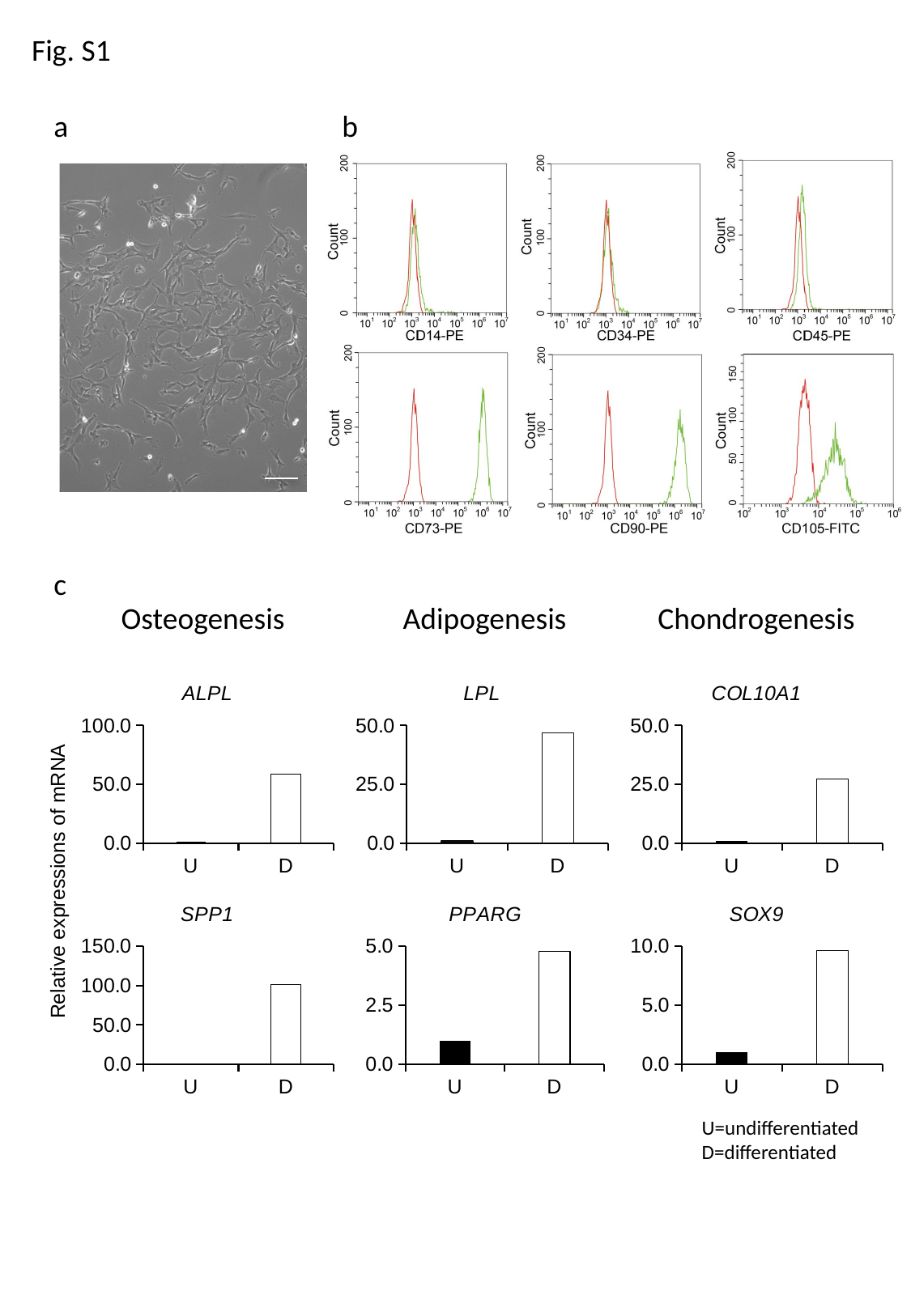

Fig. S1
a
b
c
Osteogenesis
Adipogenesis
Chondrogenesis
### Chart: ALPL
| Category | |
|---|---|
| U | 1.0 |
| D | 58.71026553764853 |
### Chart: LPL
| Category | |
|---|---|
| U | 1.0 |
| D | 46.823688725546305 |
### Chart: COL10A1
| Category | |
|---|---|
| U | 1.0 |
| D | 27.318159262491683 |Relative expressions of mRNA
### Chart: SPP1
| Category | |
|---|---|
| U | 1.0 |
| D | 101.40951128154664 |
### Chart: PPARG
| Category | |
|---|---|
| U | 1.0 |
| D | 4.772944068412976 |
### Chart: SOX9
| Category | |
|---|---|
| U | 1.0 |
| D | 9.635760978455078 |U=undifferentiated
D=differentiated
