# Supplementary material for: Prophylactic therapy with human amniotic fluid stem cells improved survival in a rat model of lipopolysaccharide-induced neonatal sepsis through immunomodulation via aggregates with peritoneal macrophages
Source: Stem Cell Res Ther. 2020 Jul 20;11:300. doi: 10.1186/s13287-020-01809-1 (PMC7370504; doi:10.1186/s13287-020-01809-1)
Supplement: Supplementary file 2 — Additional file 2: Figure S2. hAFSCs derived from two donors had similar therapeutic effects in vivo and in vitro. [file 13287_2020_1809_MOESM2_ESM.pptx]

## Slide 1
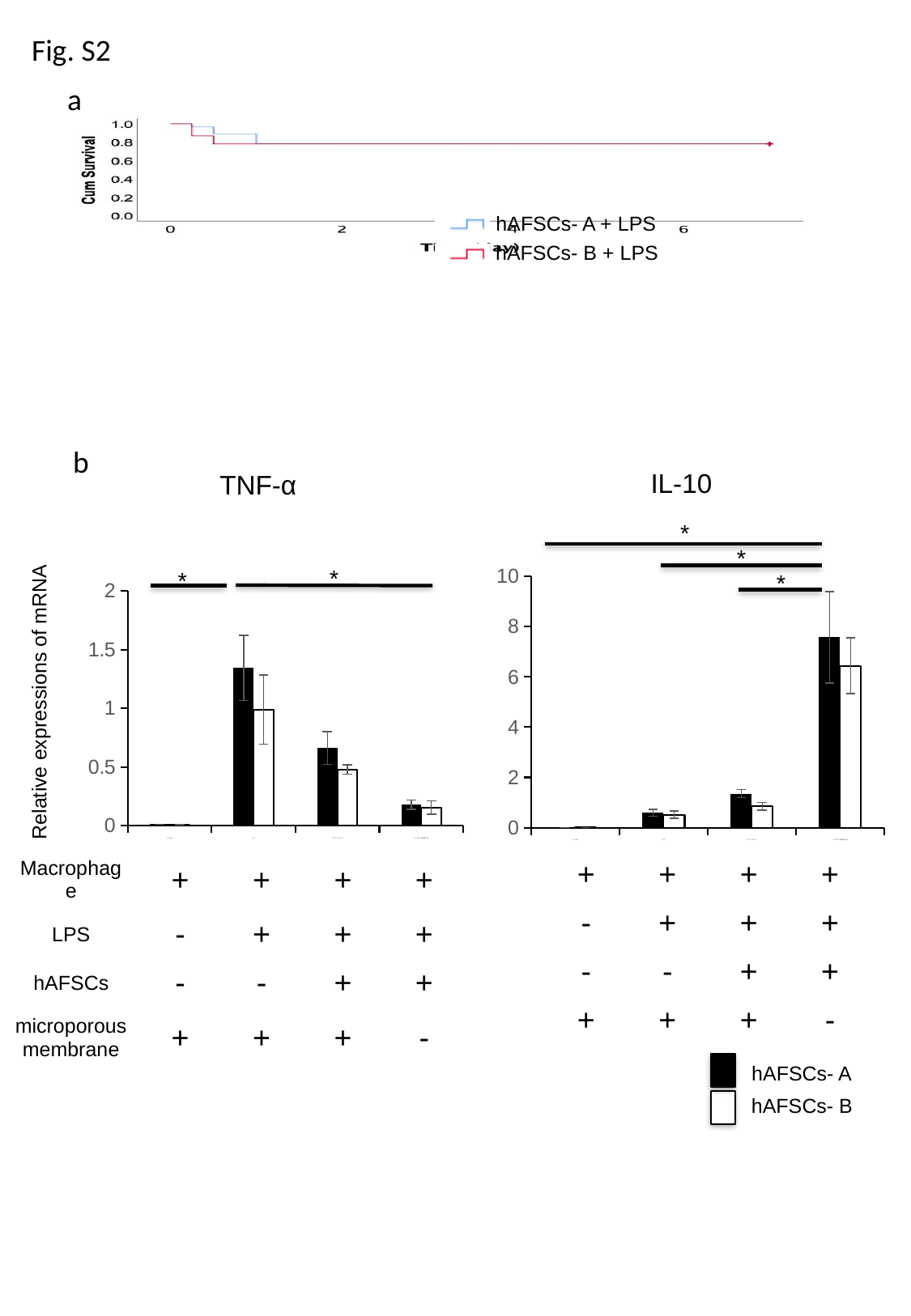

Fig. S2
a
hAFSCs- A + LPS
hAFSCs- B + LPS
 IL-10
 TNF-α
b
*
*
*
### Chart
| Category | | |
|---|---|---|
| Control | 0.012 | 0.007465 |
| LPS | 1.345 | 0.98912 |
| LPS+hAFSC | 0.661365 | 0.4792 |
| LPS/AFS_cell-cellcontact | 0.1789 | 0.1546 |*
### Chart
| Category | | |
|---|---|---|
| Control | 0.0054375 | 0.00536 |
| LPS | 0.5919 | 0.51762 |
| LPS+hAFSC | 1.3556 | 0.8489 |
| LPS/AFS_cell-cellcontact | 7.5787 | 6.44462 |*
Relative expressions of mRNA
| Macrophage | + | + | + | + |
| --- | --- | --- | --- | --- |
| LPS | - | + | + | + |
| hAFSCs | - | - | + | + |
| microporousmembrane | + | + | + | - |
| + | + | + | + |
| --- | --- | --- | --- |
| - | + | + | + |
| - | - | + | + |
| + | + | + | - |
hAFSCs- A
hAFSCs- B
