# Supplementary figures and images for: Prophylactic therapy with human amniotic fluid stem cells improved survival in a rat model of lipopolysaccharide-induced neonatal sepsis through immunomodulation via aggregates with peritoneal macrophages
Source: Stem Cell Res Ther. 2020 Jul 20;11:300. doi: 10.1186/s13287-020-01809-1 (PMC7370504; doi:10.1186/s13287-020-01809-1)

## Slide 1
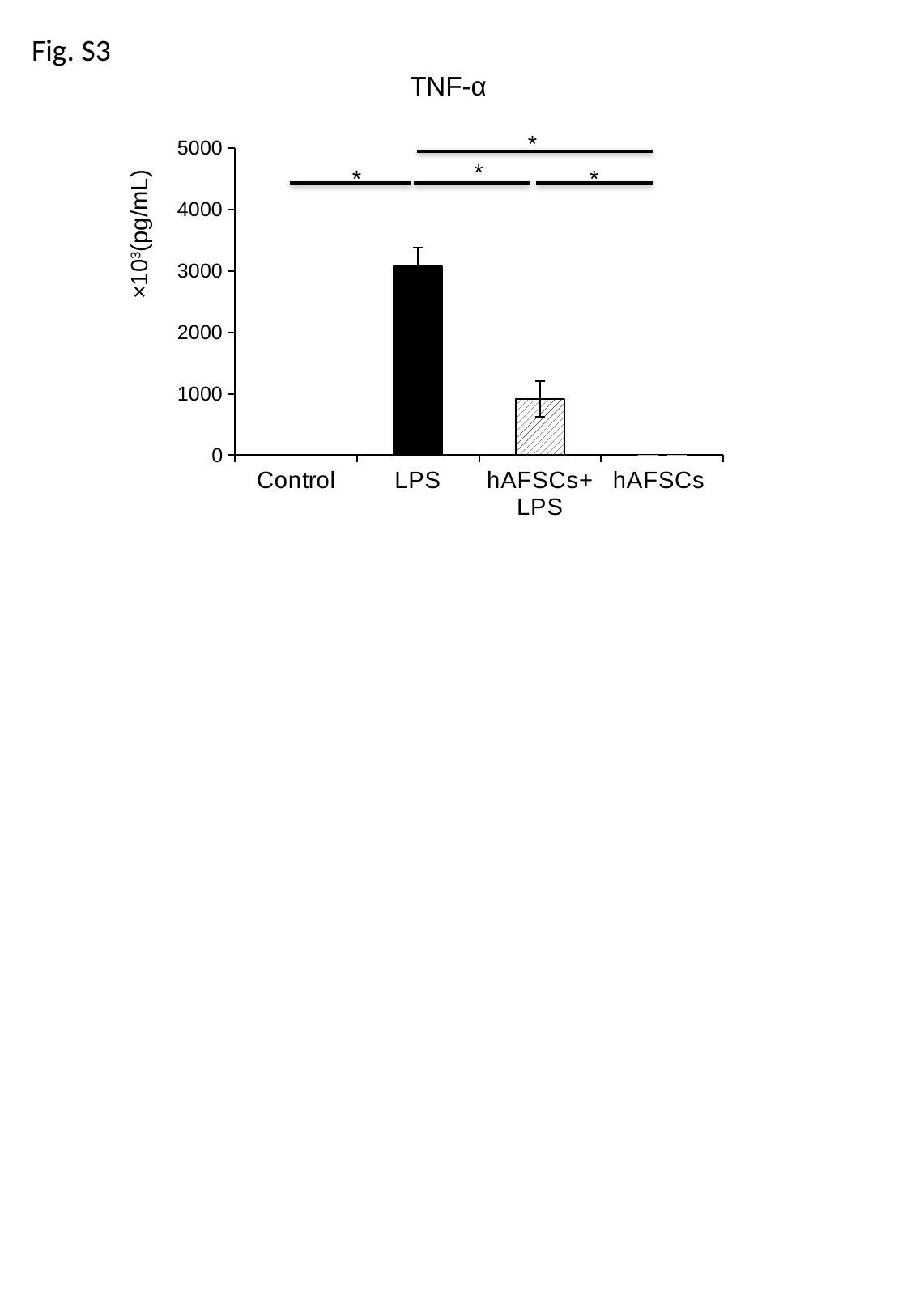

Fig. S3
TNF-α
*
### Chart
| Category | |
|---|---|
| Control | 0.6 |
| LPS | 3077.45 |
| hAFSCs+LPS | 914.55 |
| hAFSCs | 0.5 |*
*
*
×103(pg/mL)

Supplement: Supplementary file 3 — Additional file 3: Figure S3. hAFSCs alone did not increase TNF-α in serum in rats. [file 13287_2020_1809_MOESM3_ESM.pptx]

## Slide 1
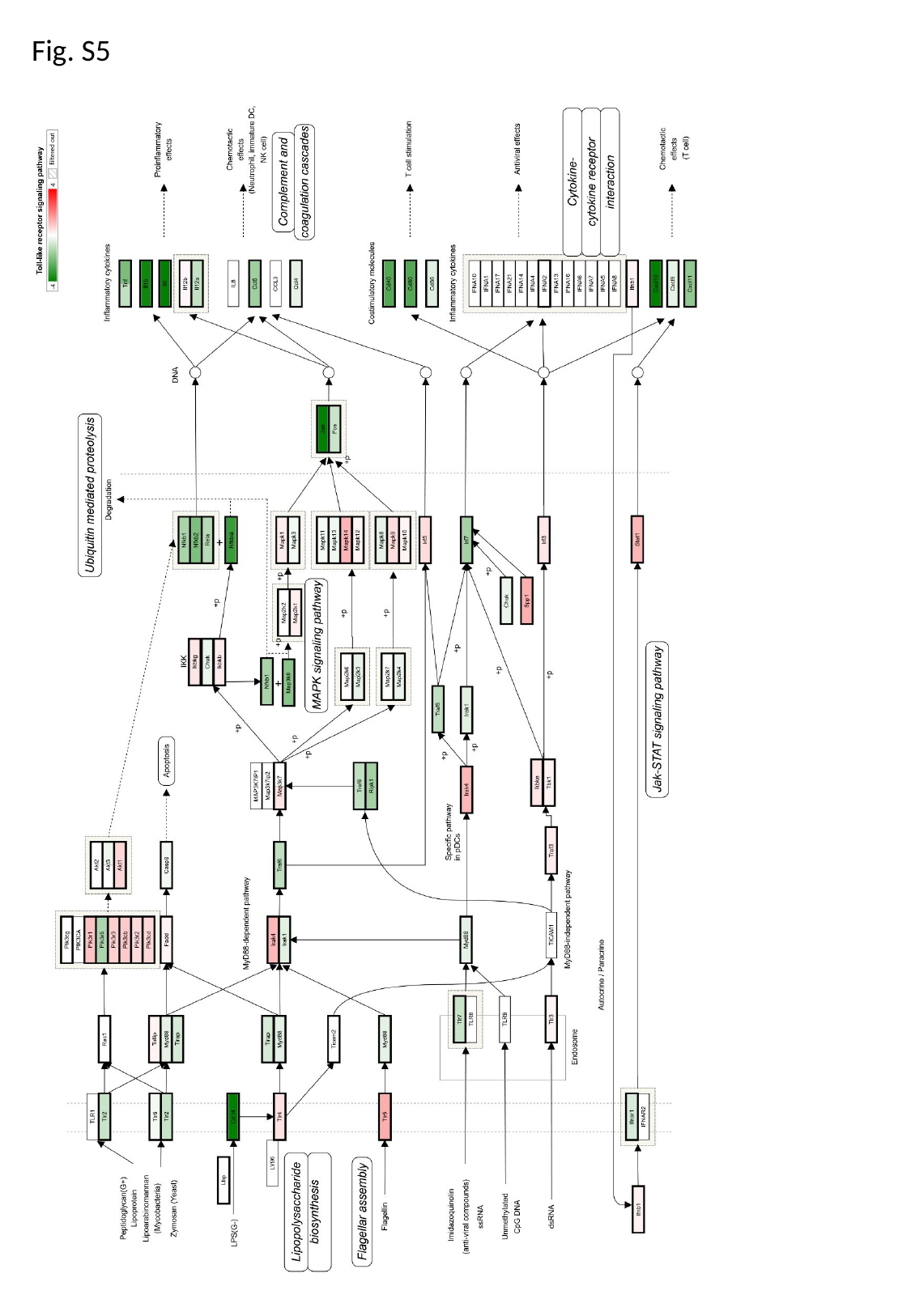

Fig. S5

Supplement: Supplementary file 5 — Additional file 5: Figure S5. Toll-like receptor signaling pathway. [file 13287_2020_1809_MOESM5_ESM.pptx]

## Slide 1
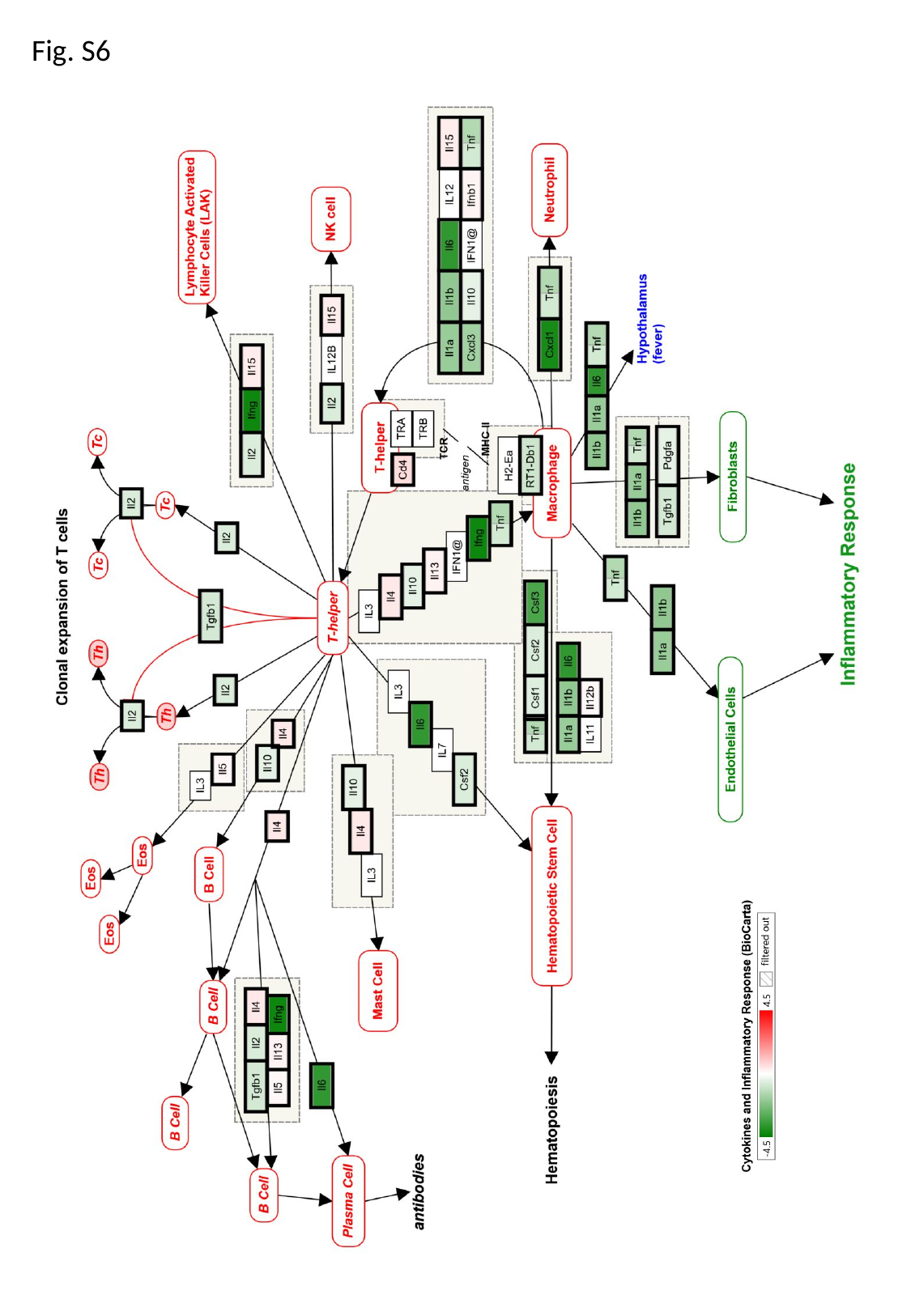

Fig. S6

Supplement: Supplementary file 6 — Additional file 6: Figure S6. The cytokines and inflammatory response pathway. [file 13287_2020_1809_MOESM6_ESM.pptx]
