# Supplementary material for: Prophylactic therapy with human amniotic fluid stem cells improved survival in a rat model of lipopolysaccharide-induced neonatal sepsis through immunomodulation via aggregates with peritoneal macrophages
Source: Stem Cell Res Ther. 2020 Jul 20;11:300. doi: 10.1186/s13287-020-01809-1 (PMC7370504; doi:10.1186/s13287-020-01809-1)
Supplement: Supplementary file 4 — Additional file 4: Figure S4. Supportive information related to macrophage phenotypic switch from M1 to M2 in cell-cell contact-independent/dependent manner at the transcriptional level. [file 13287_2020_1809_MOESM4_ESM.pptx]

## Slide 1
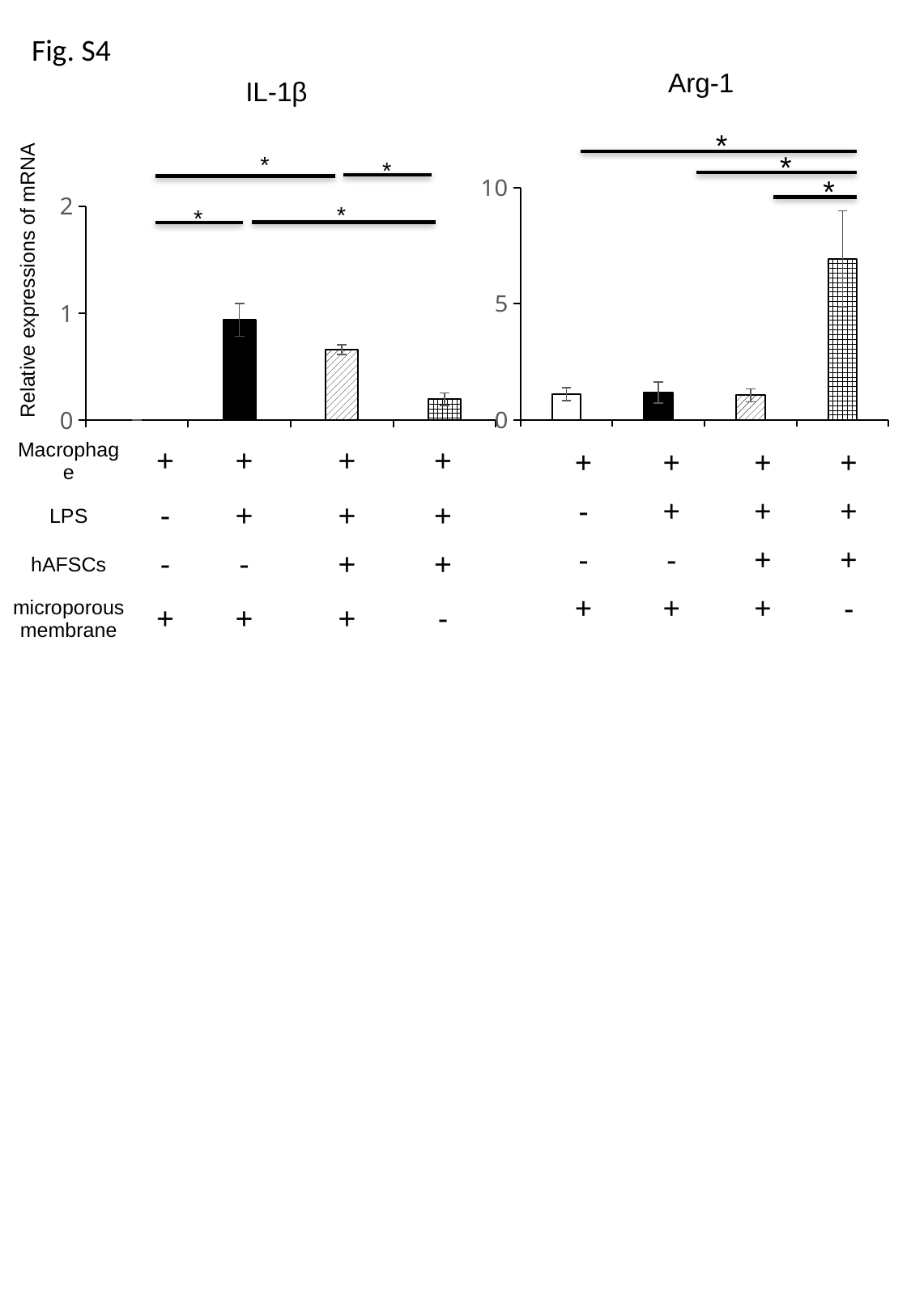

Fig. S4
 Arg-1
 IL-1β
*
*
*
*
*
*
*
### Chart
| Category | |
|---|---|
| 1 | 1.11 |
| 2 | 1.18 |
| 3 | 1.06 |
| 4 | 6.93 |
### Chart
| Category | |
|---|---|
| 1 | 0.0039 |
| 2 | 0.939 |
| 3 | 0.661 |
| 4 | 0.197 |Relative expressions of mRNA
| Macrophage | + | + | + | + |
| --- | --- | --- | --- | --- |
| LPS | - | + | + | + |
| hAFSCs | - | - | + | + |
| microporousmembrane | + | + | + | - |
| + | + | + | + |
| --- | --- | --- | --- |
| - | + | + | + |
| - | - | + | + |
| + | + | + | - |
