# Supplementary material for: Prophylactic therapy with human amniotic fluid stem cells improved survival in a rat model of lipopolysaccharide-induced neonatal sepsis through immunomodulation via aggregates with peritoneal macrophages
Source: Stem Cell Res Ther. 2020 Jul 20;11:300. doi: 10.1186/s13287-020-01809-1 (PMC7370504; doi:10.1186/s13287-020-01809-1)
Supplement: Supplementary file 7 — Additional file 7: Table S1. List of antibodies used for flow cytometry in this study. Table S2. List of primer sequences used for RT-qPCR in this study. Table S3. List of antibodies used for immunohistochemistry in this study. [file 13287_2020_1809_MOESM7_ESM.docx]

Table S1. List of antibodies used for flow cytometry in this study

| \| Species \| Antigen \| Clone \| Fluorochrome \| Manufacture \| \| --- \| --- \| --- \| --- \| --- \| \| CD14 \| Human \| HCD14 \| PE \| BioLegend \| \| CD34 \| Human \| 581 \| PE \| BioLegend \| \| CD45 \| Human \| HI30 \| PE \| BioLegend \| \| CD73 \| Human \| AD2 \| PE \| BioLegend \| \| CD90 \| Human \| 5E10 \| PE \| BioLegend \| \| CD105 \| Human \| 43A3 \| FITC \| BioLegend \| |
| --- | --- | --- | --- | --- | --- | --- | --- | --- | --- | --- | --- | --- | --- | --- | --- | --- | --- | --- | --- | --- | --- | --- | --- | --- | --- | --- | --- | --- | --- | --- | --- | --- | --- | --- | --- |

Table S2. List of primer sequences used for RT-qPCR in this study

| \| Gene \| Forward primer (5′-3′) \| Reverse primer (5′-3′) \| \| --- \| --- \| --- \| \| *β-actin* \| CTAAGGCCAACCGTGAAAAGA \| CCAGAGGCATACAGGGACAAC \| \| *TNFα* \| ATGTGGAACTGGCAGAGGAG \| ACGAGCAGGAATGAGAAGAGG \| \| *IL-10* \| AGAAGCTGAAGACCCTCTGGATAC \| GCTCCACTGCCTTGCTTTTATT \| \| *IL-1β* \| CACCTCTCAAGCAGAGCACAG \| GGGTTCCATGGTGAAGTCAAC \| \| *Arginase-1* \| GTGGCGTTGACCTTGTCTTG \| CCTGGTTCTGTTCGGTTTGC \| \| *ALPL* \| CTGGTAGGCGATGTCCTTA \| ACGTGGCTAAGAATGTCATC \| \| *SPP1* \| ATCACCTGTGCCATACCAGT \| CACATCGGAATGCTCATTGC \| \| *LPL* \| GAGATTTCTCTGTATGGCACC \| CTGCAAATGAGACACTTTCTC \| \| *PPARG* \| GCTGTTATGGGTGAAACTCTG \| ATAAGGTGGAGATGCAGGTTC \| \| *COL10A1* \| GCCCAAGAGGTGCCCCTGGAATAC \| CCTGAGAAAGAGGAGTGGACATAC \| \| *SOX9* \| AGACAGCCCCCTATCGACTTC \| TGCTGCTTGGACATCCACAC \| |
| --- | --- | --- | --- | --- | --- | --- | --- | --- | --- | --- | --- | --- | --- | --- | --- | --- | --- | --- | --- | --- | --- | --- | --- | --- | --- | --- | --- | --- | --- | --- | --- | --- | --- | --- | --- | --- |

Table S3. List of antibodies used for immunohistochemistry in this study

| Antigen | Host | Type | Dilution | Code | Source |
| --- | --- | --- | --- | --- | --- |
| GFAP | Rabbit | Polyclonal | No diluent | IS524 | Dako |
| Iba-1 | Rabbit | Polyclonal | 1:100 | 019-19741 | Wako |
| Iba-1 | Mouse | Monoclonal | 1:500 | 016-26721 | Wako |
| MPO | Rabbit | Polyclonal | 1:50 | ab9535 | Abcam |
| Human Mitochondria | Mouse | Monoclonal | 1:50 | MAB1273C3 | Sigma-Aldrich |
| CD68 | Rabbit | Polyclonal | 1:100 | bs-0649R | Bioss |
